# Eye lens opacities and cataracts among physicians and healthcare workers occupationally exposed to radiation: A systematic review and meta-analysis

**DOI:** 10.15537/smj.2022.43.7.20220022

**Published:** 2022-07

**Authors:** Ayman S. Alhasan, Waseem A. Aalam

**Affiliations:** *From the Department of Radiology and Medical Imaging (Alhasan), College of Medicine, Taibah University, Al Madinah Al Munawarah, and from the Department of Ophthalmology (Aalam), Faculty of Medicine, University of Jeddah, Jeddah, Kingdom of Saudi Arabia.*

**Keywords:** cataract, lens opacities, radiation, meta-analysis, healthcare workers

## Abstract

**Objectives:**

To evaluate the risk of developing eye lens opacities and cataracts among physicians and healthcare workers occupationally exposed to radiation.

**Methods:**

Our literature search captured articles published in Embase, Web of Science, PubMed, Cochrane Library, Cumulative Index to Nursing and Allied Health Literature, and Google Scholar databases until September 2021. Then, we retrieved articles reporting cataracts and eye lens opacities induced by radiation exposure among healthcare professionals. The outcomes of interest were cataracts, nuclear opacity, cortical opacity, posterior subcapsular opacity, and any lens opacity.

**Results:**

Of the 4123 articles identified, 15 studies met the inclusion criteria. Healthcare workers exposed to radiation had a significantly greater risk of posterior subcapsular cataracts (PSCs), cataracts, and any lens opacities than those of the non-exposed participants (*p*<0.05). The cortical opacity was not significantly different between the exposed and non-exposed participants (*p*>0.05). Radiation was not determined to be a risk factor for nuclear opacity as it was significantly greater in the control group than the exposed participants. Subgroup analysis revealed that nurses had the highest risk for PSCs (risk ratio = 4.00), followed by interventional cardiologists (risk ratio = 3.85).

**Conclusion:**

The risk of posterior subcapsular opacities and cataracts is significantly higher in healthcare workers with occupational radiation exposure than in non-exposed workers, highlighting the necessity to enhance and promote the wearing of protective measures with high safety levels.

**T**he lens of the eye is a transparent structure that is sensitive to radiation. The potential health impact of radiation on the eye lens include opacities and visual impairments in the form of cataracts.^1^ Cataracts refer to the opacification of the normal crystalline lens of one or both eyes, which alters their transparency and affects the refractive index. This leads to varying degrees of visual impairments, and consequently, decreased life’s quality.^2^ Globally, cataracts are considered the second most common cause of visual impairments, observed in 33% of cases with visual impairments.^3^ Three morphological types of cataracts have been described in the literature: cortical, nuclear, and subcapsular.^4^ The most common cataracts are nuclear cataracts that are identified by a yellowish discoloration of the middle section of the lens and sclerosis. Cortical cataracts are distinguished by white and spoke-like opacities. They affect the cortex and can spread to the periphery of the lens. Subcapsular cataracts are divided into anterior subcapsular cataracts (ASCs) and posterior subcapsular cataracts (PSCs), which affect the anterior and posterior cortex.^5^ Posterior subcapsular cataracts account for approximately 10% of all types of cataracts, and almost half of these cases occur concurrently with nuclear, cortical, or ASCs, referred to as mixed cataracts.^6^

In general, the causes of lens opacities and cataracts are multifactorial. The factors contributing to the development of cortical and nuclear cataracts have been established. In addition to age, various risk factors contribute to lens opacities and cataract formation.^7^ These causes can be genetic or congenital, or associated with disease complications (such as diabetes and glaucoma), trauma, exposure to toxic elements, or radiation.^8,9^ In this context, many studies proved that non-ionizing radiation such as Ultraviolet radiation, infrared, radiofrequency, and electric shocks may be possible causes of cataract.^10-13^

Epidemiological research and animal studies on pathological mechanisms suggested that cataract development was associated with ionizing radiation.^14,15^ Chronic occupational radiation exposure is linked to a higher incidence of cataracts.^16^ The impact of continuous ionizing radiation doses on lens alterations has been extensively studied in the medical field.^17-19^ Furthermore, many researches have described a greater prevalence of lens opacities and cataracts among healthcare professionals frequently exposed to low doses of radiation.^20^ Physicians practicing radiologic-guided interventions, such as interventional cardiologists (ICs) and interventional radiologists, are frequently subjected to ionizing radiation and are considered to have a greater risk of negative effects. Similarly, nurses and technicians working with these healthcare professionals during relevant procedures are highly exposed, especially if they adopt inappropriate protection measures. Radiation-based cataracts are directly associated with radiation dose; conversely, the latent time needed for radiation-based cataract development is conversely associated with radiation dose.^18^

Nevertheless, the lowest dose responsible for cataract formation and radiation dose-response relationships has yet to be established. Radiation-induced cataracts have been proposed to take place only following an exposure to high-dose of radiation. However, this hypothesis has been challenged by studies demonstrating that a higher risk of cataracts persists despite a low-dose radiation exposure.^21-23^ Posterior subcapsular cataracts are the most common reported lens alteration in health professionals.^19^ To decrease the prevelance of cataract among healthcare workers, the International Commission for Radiological Protection has reduced the threshold dose for radiation-induced cataract from 150 millisievert (mSv) to 20 mSv per year, averaged over 5 years with no single year exceeding 50 mSv.^24^ Consequently, the new European Directive 2013/59/Euratom (EU2013/59) updated the calssification of workers exposed to radiation and stated that workers receiving an equivalent dose to the lens more than 15 mSv/year should be classified as category A.^25^ To prevent health careworkers from exceeding the new lens dose limit, Cornacchia et al^26^ suggested to estimate the maximum number of procedures carried out by each healthcare worker and to take into account the nature and time spent.

A systematic review and meta-analysis was carried out by Elmaraezy et al^27^ and included articles issued before 2015, which evaluated the risk of developing cataracts induced by radiation among ICs and catheterization lab staff only. The latter group presented a higher risk of radiation-associated posterior lens opacity. Based on these findings, we carried out the present systematic review and meta-analysis to investigate the risk of radiation-associated eye lens opacities and cataracts among all physicians and healthcare workers occupationally exposed to radiation.

## Methods

This study was carried out following the Preferred Reporting Items for Systematic Reviews and Meta-Analyses (PRISMA) guidelines, and written according to the Meta-analysis of Observational Studies in Epidemiology proposal.^28,29^ The following PECOS question was formulated; Population (physicians and healthcare workers); Exposure (occupational exposure to ionizing radiation); Comparison (non-exposed physicians and healthcare workers); Outcome (eye lens opacities and cataracts development); Studies (cross-sectional, cohort, and case-control studies).

Both electronic and manual searches were carried out on PubMed, Web of Science, Excerpta Medica dataBASE (EMBASE), Cumulative Index to Nursing and Allied Health Literature, and the Cochrane Library databases from inception until September 2021 to identify potentially eligible articles. We developed the search strategies in collaboration with an expert academic librarian in systematic reviews who is not an author of this paper. Search strategy consisted on applying the EMBASE and Medical Subject Headings search terms when accessible. We used the following terms: “radiation or ionizing radiation” and “cataract or eye lens opacities or risk or effect” and “physicians or health care workers or cardiologists/cardiology or technologists or radiologists or gastroenterologists or orthopedic surgeons.”

### Eligibility criteria

We screened relevant articles by their title and abstract after removing duplicates. Studies were eligible for inclusion if they addressed lens opacities and cataracts in healthcare workers or physicians exposed to ionising radiation. The remaining studies were examined to evaluate eligibility.

The inclusion criteria for articles were as follows: 1) cross-sectional, cohort, and case-control studies reporting the prevalence of lens opacities and cataracts induced by radiation among physicians or healthcare workers; 2) the use of questionnaires to collect demographic information and work-related information; 3) publications reporting sufficient information to calculate risk ratios; and 4) studies published as original articles. The exclusion criteria for articles included: 1) full text not electronically accessible; 2) publication in non-English language; 3) comments, letters, editorials, protocols, guidelines, and review papers; 4) radiation exposure effects in animal studies; and 5) studies with insufficient outcome data.

We assessed the eligibility of all potential articles according to above criteria. A third author solved the disagreements, if exist, from literature screening.

After verifying the inclusion and exclusion criteria, we retrieved data from the eligible studies. We collected the following information using a standardized data sheet: 1) article ID (name of the first author, publication year); 2) article design; 3) number and age of participants (exposed and non-exposed); 4) country of study; 5) duration of occupational work; 6) lens opacity scoring system used; 7) dose of radiation; and 8) outcomes. A third reviewer was consulted to check the data sheet for accuracy. If some relevant data were not available, we contacted the corresponding author by email to request the missing data; if no response was received after a reminder, the study was excluded from the systematic-review and meta-analysis.

### Quality assessment

We evaluated the quality of the cohort and cross-sectional articles, selection bias, comparability of the exposed and control participants, and outcome evaluation following the Newcastle-Ottawa Scale (NOS). The NOS cross-sectional and cohort systems evaluated 3 sections: 1) selection of exposed and non-exposed groups (maximum of 4 and 5 points for cohort and cross-sectional studies); 2) comparability of study groups (1 or 2 points); and 3) evaluation of outcomes (1, 2, or 3 points). Quality evaluation was carried out independently. The discordance was resolved by discussion. Articles with 0 or 1 ★ in the selection section or 0 2605 in the comparability section or 0 or 1 ★ in the outcome section were assigned a poor-quality score. Articles with 2 ★ in the selection section and one or 2 ★ in the comparability section and 2 or 3 ★ in the outcome section were assigned a fair-quality score. Articles with 3 or 4 ★ in the selection section and 1 or 2 ★ in the comparability section and 2 or 3 ★ in the outcome section were assigned a good-quality score.^30^

The outcomes of interest were cataracts, nuclear opacity, cortical opacity, posterior subcapsular opacity, and any lens opacity.

### Statistical analysis

RevMan, version 5.4 (Cochrane Collaboration, Oxford, United Kingdom) was used to carry out statistical analyses. Risk ratios (RRs) and 95% confidence intervals (CIs) were calculated to assess the outcomes. A *p*-value of <0.05 was considered significant. We carried out the Cochrane Chi-squared test to estimate the heterogeneity of the articles, with a a *p*-value of <0.05 indicating the existence of heterogeneity. To estimate the impact of heterogeneity on the meta-analysis, I^2^ values were calculated. Heterogeneity values of ≥50% and *p*<0.05 indicated a moderate to high degree of heterogeneity in pooled articles. We used a fixed-effects design when I^2^ <50% with *p*>0.05; otherwise, we adopted a random-effects design.^31^ Furthermore, we carried out a sensitivity and subgroup analyses to assess the possible source of heterogeneity. We carried out Egger’s test using Statistical Package for Social Sciences, version 25 (IBM Corp., Armonk, NY, USA) to evaluate publication bias, which was further estimated by visual inspection of symmetry in the funnel plots.

## Results

**Figure 1:**
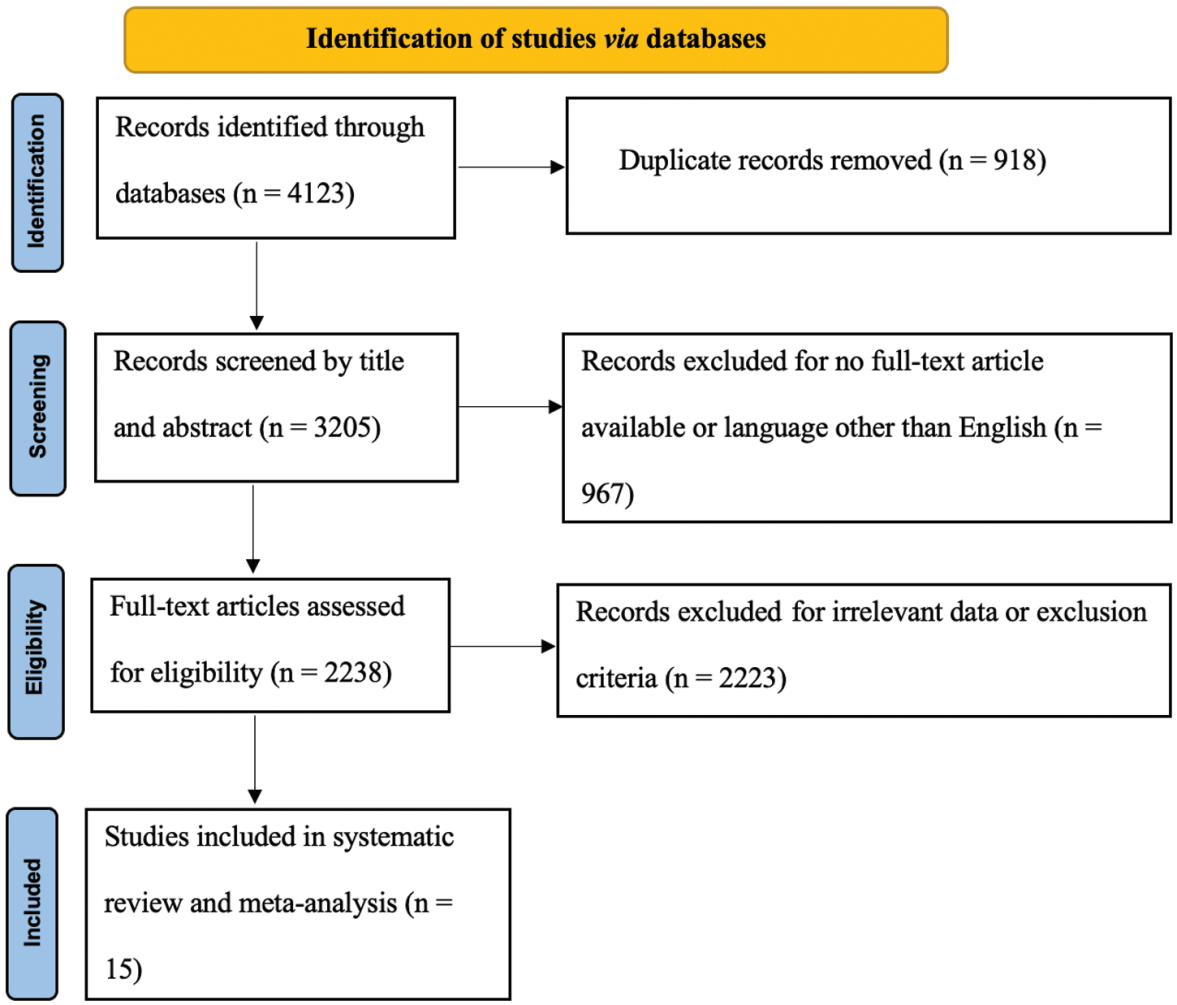
- Preferred Reporting Items for Systematic Reviews and Meta-Analyses flowchart of this meta-analysis

We identified 4123 articles for screening, of which 2238 abstracts were identified as potentially eligible and retrieved for full text review. In total, 15 articles met the eligibility criteria and were included in this study. Figure 1 presents the PRISMA flowchart.

All included articles were published between 2009-2019 and were distributed among 10 countries. Among the 15 articles included in this study, 11 were cohort studies, and 4 were cross-sectional studies. The sample sizes of the included articles varied from 37-3240 participants, and their ages ranged between 19-75 years. The duration of occupational work ranged from 3 months to 50 years. Three types of lens scoring systems were used: Lens Opacities Classification System II (LOCS II), System III (LOCS III), and Modified Merriam-Focht (MF) scoring system. Quantitative measures of radiation ranged from 0.5 μSv to 43 Sv. Table 1 describes the data extarcted from the articles used in the present meta-analysis.

**Table 1:** - List of articles used in this meta-analysis with their geographic distribution, study design, participant characteristics, and outcomes.

Study	Study design	Country	Participants (occupational category)			Age (years)	Duration of occupational work	Lens scoring system	Outcomes (lens area assessed)	Radiation dose
			n	Exposed	Non-exposed					
Andreassi et al^44^	Cohort	Italy	746	466 (218 ICs and electrophysiologists, 191 nurses, and 57 technicians)	280	35-53	5-24 years	ND	Cataract	Median lifetime effective dose physicians: 21 mSv nurses: 7 mSv
Auvinen et al^33^	Cohort	Finland	37	21 (14 radiologists, 2 interventional radiologists, 3 cardiologists, and 2 surgeons)	16 (physicians excluding radiologists and cardiologists)	45-74	At least 15 years	LOCS II	Posterior subcapsular, cortical change, nuclear opalescence, nuclear color, nuclear opacities	Mean cumulative radiation dose based on dosimeter readings: 111 mSv
Barbosa et al^34^	Cross-sectional	Brazil	200	112 ICs and healthcare workers in cardiac hemodynamics: nurse, physician, technician, or technologist	88 (cardiologists not exposed to radiation)	36-66	62% of the professionals: <20 years of work, and 31% presented between 5-10 years of work.	LOCS III	Posterior subcapsular, lens opacities, cortical cataract	ND
Ciraj-Bjelac et al^18^	Cohort	Austria	89	56 ICs and 11 nurses working in interventional cardiology	22 age- and gender-matched unexposed healthcare staff	25-64	1-33	MF scoring system	Posterior lens changes	0.01-43 Gy
Ciraj-Bjelac et al^17^	Cohort	Austria	86	52 healthcare workers in interventional cardiology	34 age- and gender-matched unexposed healthcare staff	19-67	1-20	MF scoring system	Posterior lens changes	Cumulative dose: 0.026-21 Sv
Domienik-Andrzejewska et al^45^	Cohort	Poland	147	69 (interventional cardiologists)	78 physicians, scientific/administrative staff, and nurses	37-68	5-36 years	LOCS III	Nuclear opalescence, nuclear color, cortical opacities, posterior subcapsular opacities	ICs: 224 mSv (left eye)85 mSv (right eye)
Jacob et al^36^	Cross-sectional	France	205	106 ICs	99 non-medical workers	>40	ND	LOCS III	Any lens opacities, nuclear opacities, cortical opacities, posterior subcapsular opacities	ND
Karatasakis et al^46^	Cross-sectional	USA	117	99 ICs and cath-lab staff	18 participants (industry, research staff, and others)	36-60	ND	MF scale	Any lens opacities, cortical and posterior subcapsular lens changes, Frank opacities	Cumulative radiation dose to the lens: 0.1-22.5 Gy (Mean: 4.5)
Matsubara et al^40^	Cohort	Thailand	85	Cardiac catheterization laboratories staff: 7 ICs, 41 nurses, and technologists	37 age- and gender-matched volunteers	19-59	0.4-35 years	MF scale	Posterior lens opacities	Cumulative radiation dose to the lens: 0.03-8.5 Sv
Milacic et al^47^	Cohort	Serbia	3240	1560 health workers	1680 health workers	28-52	6-26 years	ND	Cataract	Equivalent doses 0.5-8 μSv/h
Rajabi et al^48^	Cohort	Iran	95	81 interventional cardiology members (44 ICs and 37 technicians)	14 nurses not working in interventional sites	26-54	At least 4 years	LOCS III	Cataract	At least 1 mSv for at least 4 years
Scheidemann-Wesp et al^49^	Cohort	Germany	61	17 ICs	18 physicians	37-75	8-50 years	LOCS III	Nuclear opacity, cortical opacity	ND
Vano et al^19^	Cohort	Colombia and Uruguay	209	58 interventional cardiologists and 58 nurses and technicians	93 unexposed non-healthcare workers of similar age	20-69	1-40	MF scale	Posterior subcapsular lens opacities	Cumulative lens dose range: 0.1-27 Sv
Vano et al^50^	Cohort	Argentina, Colombia, and Uruguay	145	54 ICs and 69 nurses and technicians	91 non-medical professionals	20-66	4-25	MF scale	Posterior subcapsular lens changes	Cumulative eye dose range: 0.1-18.9 Sv
Yuan et al^51^	Cross-sectional	Taiwan	1866	733 cardiologists performing cardiac catheterization	988 cardiologists not performing cardiac catheterization	35-50	ND	ND	Cataract	ND

ICs: interventional cardiologists, ND: not defined, mSv: millisievert, LOCS II: Lens Opacities Classification System, version II, LOCS III: The Lens Opacities Classification System III, Gy: the gray, Sv: stroke volume, cath-lab: catheterization laboratory, MF: Modified Merriam-Focht, μSv/h: micro-Sieverts/hour

### Quality assessment

Almost half of the articles were assessed to be of good quality (n=7), while 8 were of fair quality. Tables 2 and 3 summarize the quality assessment scores for cohort and cross-sectional studies.

**Table 2:** - The quality evaluation of the cohort studies using the Modified Newcastle-Ottawa scale.

Articles	Selection			Comparability		Outcome			Quality score
	Representativeness of the sample	Selection of non-exposed group	Ascertainment of exposure	Outcome absent at the start of study	Cohort statistical analysis	Evaluation of outcome	Sufficient follow-up duration for outcomes to occur	Adequacy of follow-up	
Andreassi et al^44^	★	0	★	0	★	0	★	★	Fair
Auvinen et al^33^	★	★	★	★	★	★	★	★	Good
Ciraj-Bjelac et al^18^	★	0	★	★	★	★	★	★	Fair
Ciraj-Bjelac et al^17^	★	0	★	★	★	★	★	★	Fair
Domienik-Andrzejewska et al^45^	★	★	★	0	★	★	★	★	Good
Matsubara et al^40^	★	0	★	★	★	★	★	★	Fair
Milacic et al^47^	★	★	★	★	★	0	★	★	Good
Rajabi et al^48^	★	0	★	★	★	★	★	★	Fair
Scheidemann-Wesp et al^49^	★	★	0	0	★	★	★	★	Fair
Vano et al^19^	★	0	★	★	★	★	★	★	Fair
Vano et al^50^	★	0	★	★	★	★	★	★	Fair

★Quality score assigned for each category as per the Newcastle-Ottawa Scale criteria

**Table 3:** - The quality evaluation of the cross-sectional studies using the Modified Newcastle-Ottawa scale.

Studies	Selection				Comparability	Outcome		Quality score
	Representativeness of sample	Sample size	Comparability of non-respondents	Ascertainment of exposure	Statistical analysis design characteristics	Evaluation of outcome	Statistical analysis	
Barbosa et al^34^	★	★	★	0	★★	★	★	Good
Jacob et al^36^	★	★	★	0	★★	★	★	Good
Karatasakis et al^46^	★	★	★	★	★★	★	★	Good
Yuan et al^51^	★	★	★	0	★★	★	★	Good

★Quality score assigned for each category as per the Newcastle-Ottawa Scale criteria

Of the cohort studies (n=11), 4 scored highly in the selection domain. Sample representativeness was high, and samples were scored with a star for being somewhat representative of the target population. Exposed and non-exposed participants were drawn from the same community in 4 articles. All of the cross-sectional studies (n=4) had somewhat representative or truly representative samples. Non-response characteristics were well described for all studies. Validated measurement tools were available or described well in only one study.

Of the cohort studies, only 3 described the presence of the outcome at the beginning of the cohort study; the remaining articles scored one star. All of the studies described statistical analyses comparing exposed and non-exposed groups and used adjusted analyses. All cross-sectional studies controlled for the outcomes and additional factors (namely, age) scored 2 stars.

Of the cohort studies, only 2 did not describe the tools used for outcome assessment. All studies scored one star for follow-up with majority of the cohort after an adequate time period. All cross-sectional studies adopted validated assessment tools and used adequate and appropriate statistical analyses.

### Outcome measures

**Figure 2:**
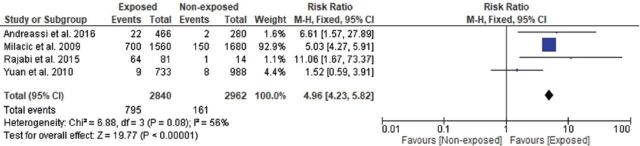
- Forest plot illustrating the results of cataract outcomes in non-exposed and exposed groups.

Four of the included studies reported the effects of occupational radiation exposure on cataract formation. As the heterogeneity was low (χ^2^=6.88, *p*=0.08, I^2^=56%), we adopted a fixed effects design. The forest plot analysis showed that cataract between the non-exposed and exposed participants was significantly different. Compared to the control group, the exposed group had approximately 5 times the risk of developing cataracts (RR=4.96; 95% CI: [4.23-5.82]; *p*<0.00001; Figure 2).

**Figure 3:**
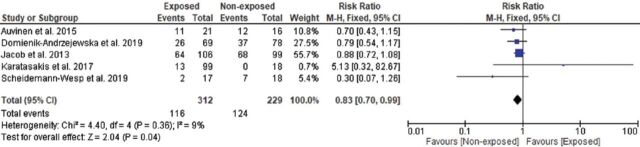
- Forest plot illustrating the results of nuclear opacity in non-exposed and exposed groups.

Five of the included studies reported the effects of occupational radiation exposure on nuclear opacity. As the heterogeneity was low (χ^2^= 4.40, *p*=0.36, I^2^=9%), we adopted a fixed effects design. The forest plot analysis demonstrated that the nuclear opacity between the non-exposed and exposed groups was significantly different (RR=0.83; 95% CI: [0.70-0.99]; *p*=0.04), indicating that radiation was not a risk factor for nuclear opacity (Figure 3).

**Figure 4:**
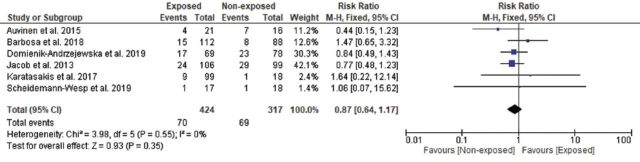
- Forest plot illustrating the results of cortical opacity in non-exposed and exposed groups.

Six of the included studies reported the effects of occupational radiation exposure on cortical opacity. As the heterogeneity was low (χ^2^=3.98, *p*=0.55, I^2^=0%), we adopted a fixed effects design. The forest plot analysis showed that the cortical opacity between the non-exposed and exposed participants was not significantly different (RR=0.87; 95% CI: [0.64-1.17]; *p*=0.35; Figure 4).

**Figure 5:**
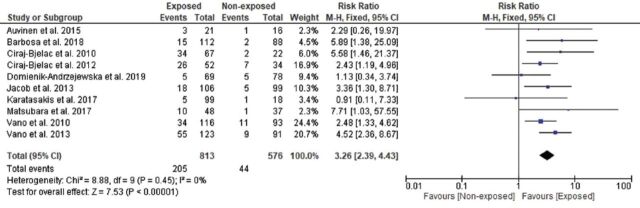
- Forest plot illustrating the results of posterior subcapsular opacity in non-exposed and exposed groups.

Ten of the included studies reported the effects of occupational radiation exposure on posterior subcapsular opacity. As the heterogeneity was low (χ^2^=8.88, *p*=0.45, I^2^=0%), we used a fixed effects design. The forest plot analysis revealed that the posterior subcapsular opacity between the non-exposed and exposed groups was significantly different. Interestingly, the exposed group had approximately 3 times the risk of developing posterior subcapsular opacity compared to the non-exposed participants (RR=3.26; 95% CI: [2.39-4.43]; *p*<0.00001; Figure 5).

**Figure 6:**
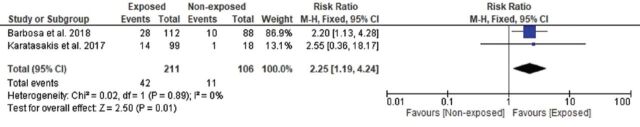
- Forest plot illustrating the results of any lens opacity in non-exposed and exposed groups.

Two of the included articles mentioned the effects of occupational radiation exposure on lens opacity (cortical and posterior subcapsular). As the heterogeneity was low (χ^2^=0.02, *p*=0.89, I^2^=0%), we adopted a fixed effects design. The forest plot analysis revealed that any lens opacity between the non-exposed and exposed groups was significantly different. Compared to the non-exposed participants, the exposed group had approximately twice the risk of developing any lens opacity (RR=2.25; 95% CI: [1.19-4.24]; *p*=0.01; Figure 6).

A sensitivity analysis was carried out to assess the origin of heterogeneity in the pooled RRs of cataract formation, nuclear opacity, cortical opacity, posterior subcapsular opacity, and any lens opacity. The outcomes did not differ substantially, indicating that the meta-analysis had strong reliability. In the leave-one-out sensitivity analysis, the RRs of cataract formation ranged from 4.15 (95% CI: [2.01-8.56]) to 5.12 (95% CI: [4.36-6.02]), nuclear opacity ranged from 0.78 (95% CI: [0.57-1.05]) to 0.86 (95% CI: [0.72-1.03]), cortical opacity ranged from 0.78 (95% CI: [0.56-1.08]) to 0.94 (95% CI: [0.63-1.39]), posterior subcapsular opacity ranged from 2.93 (95% CI: [2.06-4.16]) to 3.48 (95% CI: [2.52-4.79]), and any lens opacity ranged from 2.20 (95% CI: [1.13-4.28]) to 2.55 (95% CI: [0.36-18.17])(Table 4).

**Table 4:** - Leave-one-out sensitivity analysis of risk ratios of analyzed outcomes.

Outcomes/study excluded	Odds ratio (95% CI)	*P*-values
***Cataract formation***		
Andreassi et al^44^	4.94 (4.21-5.79)	<0.00001
Milacic et al^47^	4.15 (2.01-8.56)	0.0001
Rajabi et al^48^	4.90 (4.18-5.74)	<0.00001
Yuan et al^51^	5.12 (4.36-6.02)	<0.00001
***Nuclear opacity***		
Auvinen et al^33^	0.85 (0.70-1.02)	0.09
Domienik-Andrzejewska et al^45^	0.85 (0.70-1.03)	0.10
Jacob et al^36^	0.78 (0.57-1.05)	0.11
Karatasakis et al^46^	0.80 (0.68-0.96)	0.01
Scheidemann-Wesp et al^49^	0.86 (0.72-1.03)	0.10
***Cortical opacity***		
Auvinen et al^33^	0.92 (0.67-1.26)	0.61
Barbosa et al^34^	0.78 (0.56-1.08)	0.13
Domienik-Andrzejewska et al^45^	0.88 (0.61-1.27)	0.49
Jacob et al^36^	0.94 (0.63-1.39)	0.74
Karatasakis et al^46^	0.85 (0.63-1.15)	0.29
Scheidemann-Wesp et al^49^	0.86 (0.64-1.17)	0.34
***Posterior subcapsular opacity***		
Auvinen et al^33^	3.28 (2.40-4.47)	<0.00001
Barbosa et al^34^	3.13 (2.29-4.29)	<0.00001
Ciraj-bjelac et al^18^	3.11 (2.27-4.26)	<0.00001
Ciraj-bjelac et al^17^	3.42 (2.44-4.81)	<0.00001
Domienik-Andrzejewska et al^45^	3.48 (2.52-4.79)	<0.00001
Jacob et al^36^	3.24 (2.34-4.49)	<0.00001
Karatasakis et al^46^	3.34 (2.44-4.56)	<0.00001
Matsubara et al^40^	3.15 (2.31-4.31)	<0.00001
Vano et al^19^	3.51 (2.46-4.99)	<0.00001
Vano et al^50^	2.93 (2.06-4.16)	<0.00001
***Any lens opacity***		
Barbosa et al^34^	2.55 (0.36-18.17)	0.35
Karatasakis et al^46^	2.20 (1.13-4.28)	0.02

CI: confidence interval

A subgroup analysis for the outcome of posterior subcapsular opacity was carried out. We excluded the other outcomes due to the limited number of articles. The RRs of posterior subcapsular opacity in the exposed and non-exposed participants differed following the study design, radiation dose, period of publication, and occupational work of the participants. According to the study design, the RRs of posterior subcapsular opacity in the cohort and cross-sectional studies were similar.

When radiation dose was adopted as a moderator, the RR differed significantly between the studies. We detected the highest RR of posterior subcapsular opacity with an estimated cumulative ocular dose of ≥1 Sv (RR=3.38, *p*<0.0001). However, the exposed and non-exposed groups with lower radiation dose of <1 Sv did not reveal a notable difference (*p*=0.57). Furthermore, the RR of posterior subcapsular opacity exhibited a higher trend in studies carried out before 2015 than in those carried out after 2015. However, it was not statistically significant.

The RR of the posterior subcapsular opacity differed among healthcare workers. We observed the highest RR among nurses (RR=4.00, 95% CI: [1.41-11.30]), followed by ICs (RR=3.85, 95% CI: [2.79-5.30]), technicians and nurses (RR=2.88, 95% CI: [1.75-4.72]), physicians (RR=2.29, 95% CI: [0.26-19.97]), and technicians (RR=2.08, 95% CI: [0.96-4.50]). Moreover, we did not detect a significant difference among the technicians, physicians, and controls (*p*>0.05; Table 5).

**Table 5:** - Subgroup analyses for the risk ratios of posterior subcapsular opacity.

Subgroups	No. of studies	Risk ratio (95% CI)	*P*-values	Heterogeneity		
				Chi^2^	I^2^	*P*-values
***Study design***						
Cohort	7	3.20 (2.28-4.49)	<0.0001	6.69	10%	0.35
Cross-sectional	3	3.53 (1.70-7.31)	0.0007	2.11	5%	0.35
***Radiation dose***						
<1 Sv	2	1.36 (0.48-3.82)	0.57	0.31	0%	0.58
≥1 Sv	6	3.38 (2.37-4.82)	<0.0001	5.25	5%	0.39
***Study period***						
Before 2015	6	3.33 (2.37-4.68)	<0.0001	3.15	0%	0.68
After 2015	4	2.95 (1.42-6.11)	0.004	5.44	45%	0.14
***Occupational work***						
Physicians (radiologists, interventional radiologists, cardiologists, and surgeons)	1	2.29 (0.26-19.97)	0.45	ND	ND	ND
Interventional cardiologists	9	3.85 (2.79-5.30)	<0.0001	9.60	17%	0.29
Technicians	2	2.08 (0.96-4.50)	0.06	0.14	0%	0.70
Nurses	3	4.00 (1.41-11.30)	0.009	2.73	17%	0.30
Technicians and nurses	2	2.88 (1.75-4.72)	<0.0001	2.73	63%	0.10

No.: number, CI: confidence interval, Chi^2^: Chi-square test, Sv: stroke volume, ND: not defined

### Publication bias

**Figure 7:**
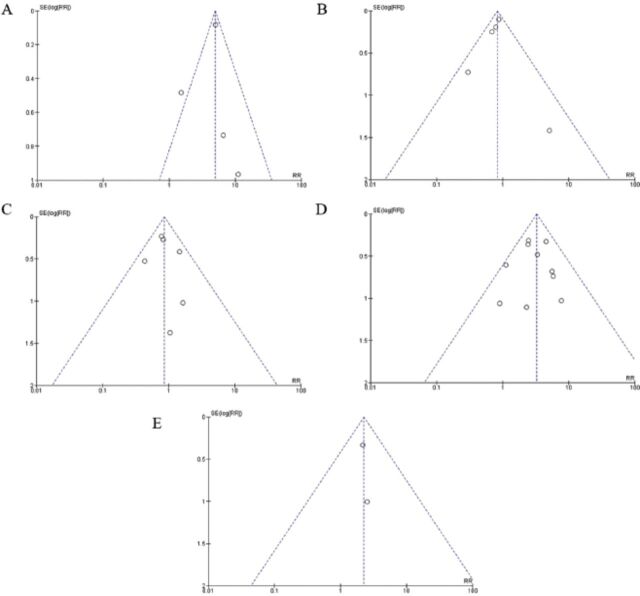
- Funnel plots demonstrating no proof of publication bias in the included articles in terms of **A**) cataract formation, **B**) nuclear opacity, **C**) cortical opacity, **D**) posterior subcapsular opacity, and **E**) any lens opacity.

Based on the Egger’s regression test as well as the visual examination of the funnel plot, we detected no proof of publication bias for any of the 5 outcomes analyzed (*p*>0.05; Figure 7).

## Discussion

The present study, comprising 15 articles assessed the risk of developing lens opacities and cataracts among physicians and healthcare professionals frequently exposed to ionizing radiation. The present meta-analysis demonstrated that physicians and healthcare professionals exposed to radiation had a notable higher risk of cataracts and PSC opacity than the non-exposed workers. These results are in line with previous reports indicating that PSC opacity is the most common kind of lens opacity linked to ionizing radiation.^32^ Auvinen et al^33^ reported that the most frequent disorder among physicians was posterior subcapsular opacity (prevalence ratio of 2.1), followed by nuclear opacity (0.82) and cortical opacity (0.41). Similarly, Barbosa et al^34^ reported an elevated incidence of any type of lens opacity (38%) followed by PSCs (13%) among healthcare professionals working in the area of cardiac hemodynamics in Brazil. The incidence of PSC opacity is 2-fold higher in occupationally exposed healthcare workers in comparison with the non-exposed workers.^17,19^ Clinical and epidemiologic research investigating radiation exposure in healthcare workers has reported the incidence of radiation-based cataracts in the healthcare field. Chodick et al^21^ identified 2382 cataracts and 647 cataract extractions among 35705 radiology technologists with an average radiation dose of 28.1 mGy. Additionally, the odds ratio of any lens opacities was 0.13 in Finnish physicians.^22^ Consequently, organizations such as the National Council on Radiation Protection and Measurements established guidelines and reports to promote awareness in health professionals and evaluate the risk of developing lens opacities due to chronic radiation exposure.^35^

Our meta-analysis demonstrated that radiation exposure did not seem to induce nuclear or cortical opacities, in agreement with previous studies. Coppeta et al^20^ demonstrated that nuclear opacity was not associated with occupational radiation exposure. The occupational lens opacities and cataract in interventional cardiology study in France revealed that nuclear and cortical opacities were not radiation-based among ICs, while the prevalence of PSCs was significantly higher.^36^ Similar findings were described in a meta-analysis carried out by Elmaraezy et al,^27^ which revealed a significantly higher incidence of PSCs among ICs but no notable difference in nuclear and cortical opacities between the exposed and control participants.

With regard to dose-response relationships, the risk of PSCs was notably higher in the ≥1 Sv group in comparison with the <1 Sv group, in agreement with precedents reports showing a notable association between occupational radiation dose and the risk of cataracts.^36^ Several epidemiological studies, animal studies, and other reports have suggested a progressive increase in cataract formation with increased doses of ionizing radiation.^24,37,38^ However, the meta-analysis of Elmaraezy et al^27^ reported contradictory results, which might be due to the limited sample size and study period (pre-2015).

A subgroup analysis was carried out to evaluate the impact of the occupation of healthcare workers on cataract development. We observed that the risk of PSCs was higher in nurses than in other healthcare professionals. Conversely, Rehani et al^39^ reported that the incidence of radiation-associated PSCs among ICs was 52% and among nurses was 45%. Similarly, Vano et al^19^ demonstrated that nearly one-third of ICs presented with high rates of PSCs after 30 years of work. In contrast, this meta-analysis demonstrated that nurses presented with a higher risk of PSCs than that of ICs. This is expected considering nurses stand next to the patient’s bed for a long time without a protective screen, in contrast to ICs. As such, nurses are highly exposed to radiation.^40^ Accordingly, this meta-analysis highlights the need to adopt adequate strategies to decrease radiation doses, ensure adequate protection of all healthcare workers, and promote the implementation of more appropriate radiation protection measures that encompass all staff members in the interventional room.

### Study strengths and limitations

In this study, a literature search was carried out using 5 different databases. The major strength of the present study is the considerable scale of included articles and the large number of participants analyzed. Unpublished articles were not included. However, the funnel plot did not reveal a publication bias. Another major strength of our meta-analysis is the high methodological quality of the included articles, which presented with good or fair-quality scores. Furthermore, radiation dose spanning the entire career of the participants was available for the majority of studies, which permitted dose-response analysis. Notably, this meta-analysis did not detect statistical heterogeneity, indicating that all studies demonstrated the same effect. Most studies (12/15) used the validated lens opacity grading systems (LOCS II, LOCS III, and MF scale), which ensured standardized outcome assessments. However, the use of different classification systems constituted an important source of discrepancies in this meta-analysis. The MF system is specifically applicable for posterior lens opacities, while LOCS III evaluates any type of cataracts.^41,42^ These grading systems also employ different methods in evaluating opacities, including retro-illumination, Scheimpflug imaging, and dilated slit-lamp biomicroscopy.^43^ These differences constitute a significant limitation when comparing the results of different studies and consequently increase the complexity of pooled analysis. Therefore, cataract classification systems should be standardized for an effective assessment of lens opacities.^43^

In conclusion, the present meta-analysis revealed a significantly higher risk of posterior subcapsular opacities and cataracts among healthcare workers occupationally exposed to radiation, suggesting that radiation exposure levels are correlated with a greater risk of cataracts. This meta-analysis indicated that nurses and ICs occupationally exposed to radiation are more prone to developing lens opacities, which reinforces the urgent requirement of strict compliance with the wearing of protective tools to minimize lens exposure to radiation. Also, a medical surveillance program should be implemented to systematically assess and detect early signs of adverse health effects among healthcare workers potentially exposed to ionizing radiation in the course of their employment.
